# Unraveling the Influence of the Anchored Headgroup and Ligand Tail Group Length on the Self-Assembly of ZnO NCs at the Air–Water Interface

**DOI:** 10.1021/acsami.5c07324

**Published:** 2025-06-10

**Authors:** Aleksandra Borkenhagen, Małgorzata Wolska-Pietkiewicz, Ilona Binkiewicz, Łukasz Richter, Rafał Zbonikowski, Jan Paczesny, Janusz Lewiński

**Affiliations:** † 119463 Institute of Physical Chemistry PAS, Kasprzaka 44/52, 01-224 Warsaw, Poland; ‡ Faculty of Chemistry, 49566 Warsaw University of Technology, Noakowskiego 3, 00-664 Warsaw, Poland

**Keywords:** zinc oxide, nanocrystals, self-assembly, organic ligand, interdigitation, Langmuir films

## Abstract

Understanding the self-assembly of nanocrystals into ordered superlattices is fundamental for tailoring nanoscale assembly and developing functional materials. However, the rational-by-design hierarchical assembly is complex, as it needs to orchestrate several physical and chemical processes. In this work, we investigate how the character of a ligand shell, particularly the type of anchored headgroup and the length of the ligand tail group, affects the self-assembly properties of nanoscale building blocks and the properties of corresponding thin films. Thus, a series of colloidal zinc oxide nanocrystals (ZnO NCs) coated with different organic shells are prepared by a self-supporting organometallic approach (OSSOM) and then self-assembled at the air–water interface. We demonstrate that ligands bearing fewer and longer alkyl chains attached to the headgroup promote a higher level of ligand interdigitation. This, in turn, leads to stronger interparticle interactions and enhanced viscoelastic properties of the resultant films. Shorter ligands and a more compact capping layer (more aliphatic chains per ligand) prevent interdigitation, allowing ZnO NCs to retain their individual character and form more elastic films. Our findings reveal the importance of organic shell design in the self-assembly of nanocrystals due to the steric control, and allow an extra level of tailorability to obtain the desired properties for the material called “superlattices” or “NC solids”.

## Graphical abstract

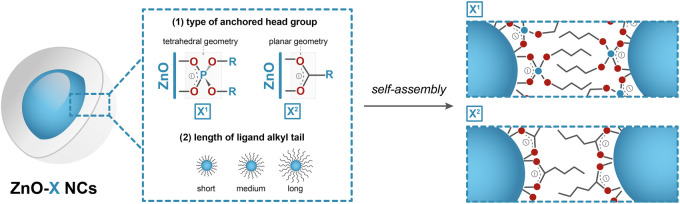

## Introduction

Colloidal nanocrystals (NCs) are important due to their remarkable tunability and functionalities. These hybrid objects are composed of an inorganic core coated with an organic shell in which the properties of both components determine the characteristics of the entire system.^1^ The organic ligands not only passivate the surface defects and provide colloidal stability but often act as factors determining the interparticle interactions and the assembly of NCs.^2^ The self-organization of colloidal NCs offers additional nanostructure tailorability to achieve properties differing strongly from their single nanoscale building blocks, and this topic attracts attention at the level of basic and applied research.^3–8^ Ordered structures called “superlattices” or “NC solids” can be achieved upon slow evaporation of carrier solvent to form thin films^9^ or by gentle destabilization of the colloidal solution to create assemblies, often distinguished as supercrystals or superparticles.^10–13^ Nonetheless, it remains highly challenging to precisely manipulate individual NCs toward well-defined hierarchical assemblies due to their polydispersity, heterogeneity, and complex nanoscale interaction forces.^3,14–16^ The self-assembly processes of NCs are affected by short-range interactions between the ligand shells, including van der Waals forces between aliphatic chains, steric hindrance, and, where applicable, hydrogen bonding interactions. These interactions modulate the core-to-core forces and collectively determine the resulting superlattice morphology.^17,18^ The arrangement of presynthesized NCs can proceed spontaneously by their self-organization without any external stimulus or with an external trigger.^19^ Notably, most of the research focused on the assembly of metal nanoparticles supported by L-type ligands, which featured the disordered and dynamic nature of the surface.^20^ In the classification proposed by Owen, ligands are categorized into three main types, i.e., X, L, and Z, based on their binding mode to nanocrystal surfaces and the number of electrons they contribute. X-type ligands are singly charged anionic donors, L-type ligands are neutral two-electron donors, and Z-type ligands are neutral two-electron acceptors.^21^

More than two decades ago, the first report on the formation of superlattices harnessing semiconductor NCs, namely, CdSe covered by trioctylphosphine oxide (TOPO) as an L-type ligand, was reported.^8^ This area of research is developing to a lesser extent. It should be noted that the highly dynamic L-type ligation might cause diffusion of coating ligands from the surface or adjust their position due to external factors, and the presence of unbound ligands in colloidal suspension can play a significant role in determining NC superlattices.^22^ In the case of widely used long-chain amines, an ordered assembly may be affected by the reaction of the surfactant with ambient CO_2_.^23,24^ So, in many instances, the pristine protective ligands are unsuitable for superlattice growth and must be replaced by other surface-binding organic ligands better suited for self-assembly processes. Undoubtedly, the NC building blocks’ chemistry–structure–performance relationship is a pivotal element in the assembly of NCs. Thus, there is a constant call for molecular-level understanding of both NCs’ surface and interface chemistry.^25,26^

Pertinent to the system considered in this work, NCs capped by X-type organic ligands, i.e., the strongly anchored monoanionic ligands, are an exciting system for studying the factors controlling self-organization processes. However, systematic studies on the influence of the character of a ligand shell, especially the type of anchored headgroup and the length of the ligand alkyl tail, on the self-assembly properties of NCs are relatively scant. Among semiconductor NCs, an interesting example represents a series of works on tuning the character of X-type capping ligands, particle size, and ligand coverage on PbS NCs.^27–30^ More recently, our group has developed a one-pot self-supporting organometallic (OSSOM) procedure for the synthesis of high-quality colloidally stable quantum-sized ZnO NCs coated with an “impermeable”^31^ and highly ordered organic shell^32,33^ composed of the X-type ligands like carboxylates,^34–36^ aminoalkoxides,^37^ phosphates,^32^ phosphinates^38^ and benzamidinates,^33^ and thoroughly exploited their properties. For example, the resulting NCs essentially exhibit a small negative impact on mammalian cell lines. However, there is a subtle interconnection between the inorganic core-organic shell dimensions and the toxicological profile.^39,40^ The effective surface passivation and the impermeable organic shell also paved the way for very efficient phase transfer of hydrophobic ZnO NCs coated with carboxylate ligands to water using the postsynthetic functionalization via the Cu­(I)-mediated azide–alkyne cycloaddition while retaining the photoluminescent (PL) properties of the NCs.^35,41^ Remarkably, the aliphatic chain length with alkyne termini of the carboxylate-capping ligand strongly affected the properties of the NCs. The Cu­(I)-mediated click chemistry functionalization of presynthesized ZnO NCs capped by 5-hexyne carboxylate with hydrophilic 2-[2-(2-azidoethoxy)­ethoxy]­ethanol triggered an effective phase transfer process. In contrast, similar attempts involving 10-undecanoic carboxylate-capping ligands were unsuccessful. Regarding the commonly observed luminescence quenching of quantum-sized semiconductor crystals upon their aggregation in an aqueous medium,^42^ the resulting water-suspendable ZnO NCs with surface-binding soft ligands exhibited the unprecedented for II–VI semiconductor nanomaterials—the aggregation-induced emission enhancement effect.^41^

Controlling the organization of NCs into ordered superstructures is crucial for technological development. Fabricating NC superlattices at the liquid surface is a promising method for the formation of self-assembled films. Therefore, we were also curious about self-assembly properties at the air–water interface of organometallic-derived ZnO NCs coated with strongly bound X-type ligands as a function of the organic ligand shell. To this end, initially, we studied ligand-induced self-assembly of ZnO NCs coated with carboxylate anchored liquid crystalline organic ligands upon compression at the air–water interface^34^ and attempted the deposition of photoactive freely suspended and free-standing films of a macroscopic area composed of various carboxylate ligand-coated ZnO NCs.^36^ Herein, to verify the effect of the character of ligand shell, including both the anchored headgroup and the length of ligand alkyl tail on the self-assembly of ZnO NCs, we report on (i) the preparation and characterization of a series of quantum-sized ZnO crystals coated with various monoanionic diorganophosphate and the respective carboxylate ligands using the OSSOM approach, and (ii) their propensity to form thin films at the air–water interface (Scheme 1).

**Scheme 1:**
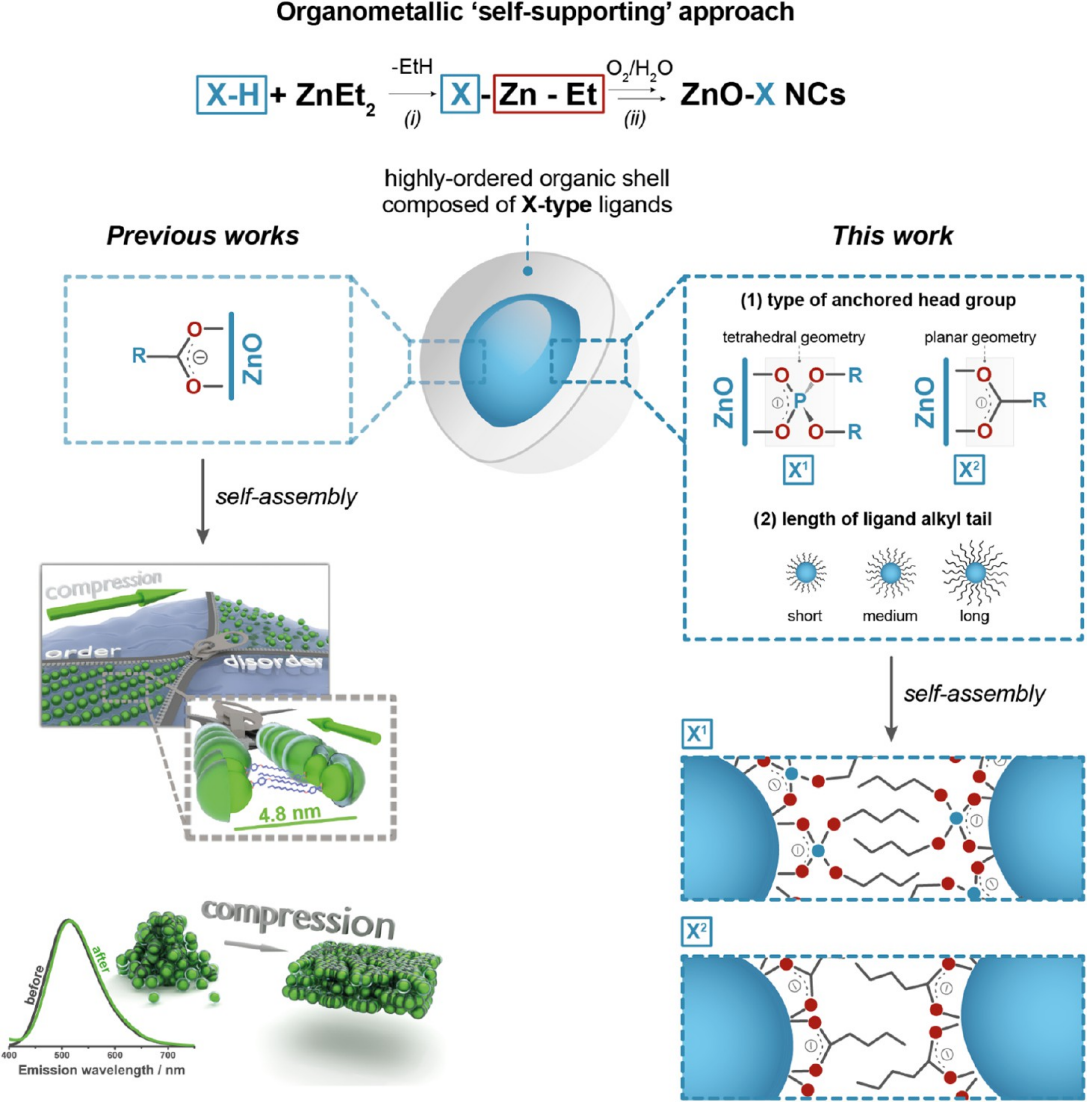
Schematic Representation of an OSSOM Procedure for the Preparation of ZnO NCs Coated with the Selected X-Type Organic Ligands and the Self-Assembly Properties of the Respective NCs at the Air-Water Interface in Previous Works *(left;* adapted with permission from ref 34, Copyright 2015 John Wiley and Sons, and with permission from ref 36, Copyright 2016 American Chemical Society) and this work *(right).*

## Experimental Section

### General Remarks

All manipulations involving diethylzinc were conducted under a dry, oxygen-free nitrogen atmosphere using standard Schlenk techniques. Tetrahydrofuran (THF) was dried and distilled with an MBraun SPS solvent purification system prior to use. All reagents were used without further purification and were purchased from commercial sources as follows: dimethyl phosphate (ABCR, Germany), dibutyl phosphate (Sigma-Aldrich), didecyl phosphate (TCI, Japan), dihexadecyl phosphate (Sigma-Aldrich), heptanoic acid (Sigma-Aldrich), undecanoic acid (Sigma-Aldrich), heptadecanoic acid (ABCR, Germany) and Et_2_Zn (ABCR, Germany). Chloroform (POCH, Poland), used for preparing solutions applied to the air–water interface, was of high-performance liquid chromatography (HPLC) grade. Ethanol (POCH, Poland) was used to clean the trough and the barriers, and all other solvents were of analytical grade. Ultrapure water characterized by surface tension of 72.75 mN·m^–1^ at 20 °C and resistivity of 18.2 MΩ·cm, used as a subphase in the Langmuir trough, was obtained from a Milli-Q water purification system.

### Preparation of Ligand-Coated ZnO NCs

ZnO NCs were prepared using the recently developed one-pot, two-step OSSOM method.^32,34,35,37–40^ In the first step, Et_2_Zn in hexane (0.5 mL of a 2 M solution in hexane, 1 mmol) was added dropwise to a solution of 1.0 mmol of selected proligand, i.e., diorganophosphate (X^1^-H) or carboxylic acid (X^2^-H), in THF (10 mL) at −78 °C, and then the reaction mixture was allowed to warm to room temperature and stirred for 24 h. Then, the solution of the in–situ–generated ethylzinc precursor in THF was exposed to air and stirred for 6 to 8 days at ambient conditions (22–27 °C). For the dimethyl phosphate proligand, the reaction mixture containing ZnO-X^1–0^ NCs formed a nondispersible precipitate. In all other cases, the mixtures remained clear and colorless, consistent with the formation of stable ZnO NCs dispersions in THF. For physicochemical characterization, the as-synthesized ZnO NCs were separated from the resulting THF solution using hexane, centrifuged (9000 rpm, 10 min), and rewashed two times with THF (2 mL).

### Langmuir and Langmuir–Blodgett Films

The air–water interface provides a well-established and deliberate platform for probing the 2D organization of amphiphilic or surface-functionalized NCs. Langmuir trough techniques enable fine control over interparticle spacing and surface pressure, offering tunability that is not accessible in bulk solvent-based self-assembly. While the aliphatic ligands on NCs are indeed poorly solvated in water, this environment promotes lateral confinement. It enhances interactions between particles, thus serving as a sensitive platform for evaluating the effects of the ligand structure on assembly behavior.

A classical analogy can be drawn to the long-standing study of fatty acids and other amphiphiles at the air–water interface, where hydrophobic tails are exposed to air, and polar heads are immersed in water. These systems do not collapse immediately upon compression but exhibit smooth phase transitions from gas-like to liquid-expanded and eventually solid-condensed monolayers. Similar principles govern NC organization at the interface, where steric and enthalpic factors dictate the monolayer stability.

The extent to which NCs are immersed in the water subphase is governed by their surface chemistry. According to wetting angle theory, more hydrophobic particles sit higher at the interface and are less submerged.^43^ The water surface remains flat for small particles (<5 μm), and capillary interactions are negligible.^44^ As such, most ligands reside in a nonaqueous environment, validating the analogy to classical amphiphiles and supporting the use of interfacial compression to probe packing behavior.

Experiments were performed with the use of a high aspect ratio (5 cm per 75 cm) Langmuir trough (NIMA, UK) equipped with surface pressure and potential sensors (Treck, Inc.), a dipper, a temperature control unit (Thermo Scientific Neslab RTE-7), and an NFT MiniBAM Brewster angle microscope (NFT NanofilmTechnologie GmbH, Germany). The BAM images represent the area with dimensions of 6.4 mm by 4.8 mm (8.3 μm per pixel). The system was placed on an active antivibration table (Standa, Lithuania) and closed in a poly­(methyl methacrylate) box to protect films against dust and air currents. Single-use filter paper strips were utilized in the surface pressure sensor and calibrated before each experiment. Before each experiment, the trough was carefully cleaned with ethanol and rinsed with water. Any remaining impurities floating on the subphase were removed in the iterative process of bringing the barriers together (resultant surface area of around 20 cm^2^) and removing the surface layer of water from in-between the barriers with an under-pressure aspirator. The temperature of the subphase was measured with two Pt100Ω resistance thermometers immersed at both ends of the trough and connected to a multimeter (Keithley 196). Studied suspensions of ZnO nanocrystallites in THF were carefully spread onto the water’s surface using a micro syringe (Hamilton). The solutions were deposited at the maximal distance between the barriers, i.e., the maximal surface area of the trough. Recently, Nguyen and Weimer showed that the procedure of deposition of the material onto the air–water interface might have an impact on the film structure. Following their guidelines, we utilized the so-called ‘touch method’.^45^ In the touch method, a droplet of nanoparticle dispersion is brought close to the air–water interface until a meniscus forms and contacts the subphase. The solution is then gently allowed to transfer to the surface either passively (by capillary capture) or actively (by slightly pushing the droplet toward the interface) without letting it fall or splash. This controlled contact minimizes disturbances to the subphase and enables more uniform film spreading compared to that of conventional drop-casting.

The experiments started around 30 min after the suspension was applied to the interface. The time delay allowed the solvent to evaporate and the system to equilibrate. Afterward, the surface area of the trough was reduced by a slow, uniform motion of the barriers with a speed of 10 cm^2^·min^–1^. To determine the contact cross-sectional area (CCSA) values, we drew a tangent line to the linear portion of the π–A isotherm between approximately 1 and 5 mN·m^–1^, where the surface pressure increased steadily. This region corresponds to the onset of significant interparticle interactions and is chosen to avoid initial noise at very low pressures and nonlinear behavior closer to film collapse. This approach ensures consistency across samples and reflects the early-stage compressibility of the monolayer prior to aggregation or collapse.

The oscillatory barrier method was used to determine the dilatational viscoelastic properties of films at the air–water interface. Films were subjected to small periodic compressions and expansions, and the changes in the surface pressure were monitored. The monolayer was initially compressed to around 2 mN·m^–1^ (collapse surface pressure was not achieved), relaxed (to 0 mN·m^–1^), and left at maximal distance between the barriers for 30 min. Afterward, the film was compressed to a target pressure. Oscillations were performed from the lowest frequency (around 0.02 Hz) to the highest frequency (approximately 0.15 Hz). The induced changes in the surface area ranged from about 1% to around 1.5% for low and high surface pressure, respectively. Ten oscillation cycles were recorded for each frequency. There was a 60 s interval between oscillation cycles.

The films were transferred onto silicon wafers using the Langmuir–Blodgett technique with a dipper speed of 10 mm·min^–1^. Ligand-coated ZnO NCs, ZnO-X^1–1^ - ZnO-X^1–3^, thin films were transferred to silicon wafers at surface pressures of 6, 9, and 10 mN·m^–1^, respectively. The Institute of Electronic Materials Technology (Warsaw, Poland) provided the silicon wafers used for the experiments. The wafers were cleaned with acetone, dipped in nitric acid (30% solution) for 30 min, and rinsed several times with water. The solid substrate was immersed in the subphase before spreading the nanocrystallite suspension onto the air–water interface. Transfer onto a solid substrate was always performed during the upstroke.

### Characterization Techniques

The size and shape of the NCs were examined using a Cs-corrected scanning transmission electron microscope (STEM HITACHI HD2700, 200 kV). Samples were prepared by slow evaporation of a droplet of a ZnO NCs colloidal solution (in THF) deposited on a holey carbon-coated 300 mesh copper TEM grid (Quantifoil). The QDs’ size distributions were determined by measuring a minimum of 100 separated nano-objects using the ImageJ program (freeware).

Powder X-ray diffraction data were collected on a PANalytical Empyrean diffractometer with Ni-filtered Cu Kα radiation (*λ* = 1.5406 Å), a secondary graphite (002) monochromator, and an RTMS X’Celerator (Panalytical, UK) with 40 kV voltage and 40 mA current and Bragg–Brentano geometry with a beam divergence of 1°. Diffraction patterns were measured in the range 2*θ* = 5 to 80° by step scanning with a step of 0.02°.

Fourier-transform infrared (FTIR) spectra were measured with a Bruker Tensor II spectrometer equipped with an attenuated total reflection (ATR) accessory.

The solvodynamic diameter of ZnO NCs was determined by dynamic light scattering analysis (DLS) with a Malvern Zetasizer Nano Z instrument (UK). A solution of ZnO NCs was filtered before analysis through a 0.45 μm filter to remove dust particles. Mean diameter values were obtained from three different runs.

Thermogravimetric analysis (TGA) was carried out with a TA Instruments Q600 instrument under a flow of artificial air to a maximum of 600 °C at a heating rate of 5 °C·min^–1^ (flow rate of 100 mL·min^–1^). Open alumina crucibles 5 mm in diagonal were used.

Optical absorption (UV–vis) spectra of the ZnO NCs colloidal solutions were collected with a Hitachi U-2910 spectrophotometer. A standard quartz cell (Hellma, Germany) with a path length of 10 mm was used and rinsed with THF before each run. PL measurements were carried out with a HITACHI Fluorescence Spectrophotometer F-7000. Both UV–vis and PL spectra were recorded under ambient conditions.

X-ray reflectometry (XRR) studies were performed on a Bruker D8 Discover diffractometer with a Cu Kα radiation source. A parabolic Goebel mirror formed a monochromatic parallel beam. The system was equipped with an Eulerian cradle and reflectometry sample stage, which ensured precise sample positioning. A scintillation counter and an automatic absorber on the primary beam allowed for a linear dynamic range greater than 10^8^ cps. The “box model”, in which the studied film was subdivided into slabs of various electron densities, was used for the fitting procedure. The starting parameters were roughly calculated according to the CPK model (named after the names of Corey, Pauling,^46^ and Koltun^47^) in the case of ligands and based on other experimental methods for ZnO cores. This model allows for the simple estimation of the dimensions of molecules based on their geometry. Fitting was performed by using the Leptos 4.02 software package provided by Bruker.

Scanning electron microscopy (SEM) images were taken using a NovaSEM instrument (FEI). The Langmuir–Blodgett samples transferred onto silicon wafers (as described in the previous section) were left to dry in air. Such prepared samples were immobilized in the SEM stages with a silver paste.

## Results and Discussion

### Preparation and Physicochemical Characterization of Ligand-Coated ZnO NCs

The preparation of high-quality, ultrasmall, and isotropic ZnO NCs has recently advanced significantly due to the introduction of a highly promising one-pot self-supporting organometallic (OSSOM)^32,35,37–40^ procedure via the controlled exposition of a [EtZn­(X)]-type molecular precursor (where X = monoanionic organic ligand; abbreviated herein as X^1^ for diorganophosphate and X^2^ carboxylate-type ligands, respectively) solution to atmospheric air (where both H_2_O and O_2_ act as oxygen sources) (Figure 1). Notably, the OSSOM-derived NCs display an irreversibly anchored shell, which ensures that the NC properties remain unaltered during postsynthetic modifications and further applications.^32,35,40,41,48^ Thus, the possibility of preparing well-defined, stable ZnO NCs with well-anchored ligands opened the possibility of studying the effect of organic ligand shell structure on the self-assembly of these nanoscale building blocks within thin films, according to the Langmuir–Blodgett method.^34,36^

**Figure 1:**
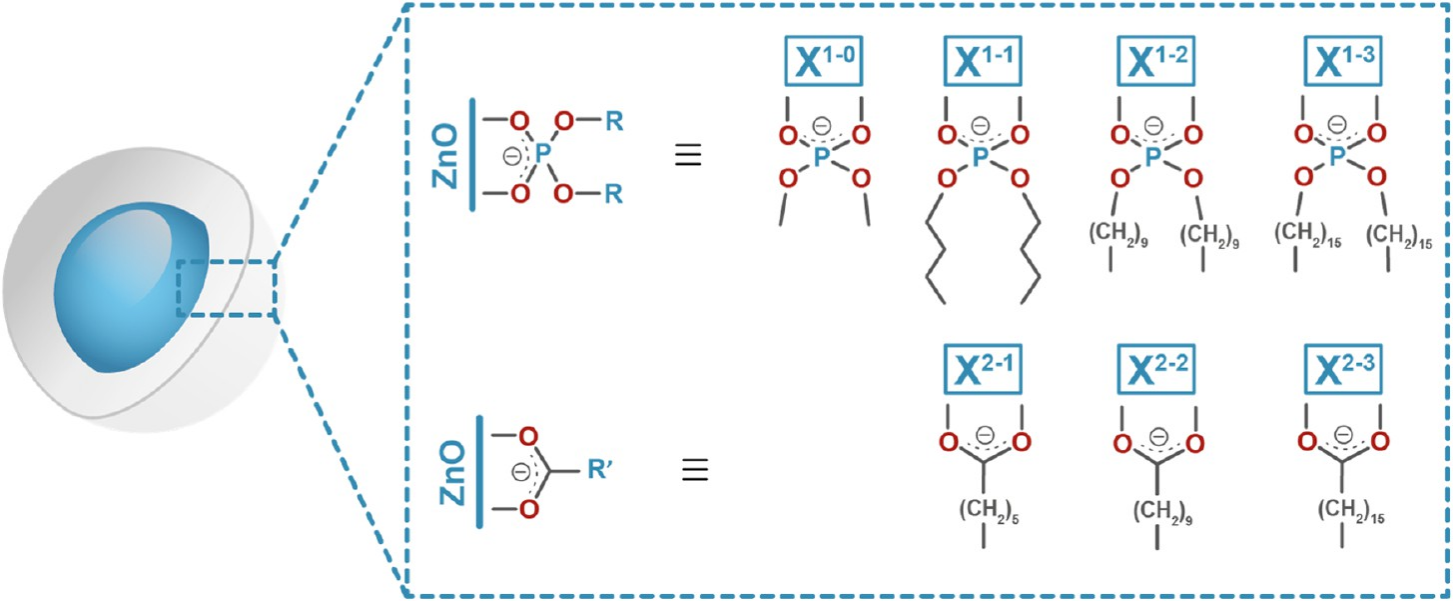
Schematic representation of ligand-coated ZnO NCs coated with selected diorganophosphates (X^1^) or carboxylates (X^2^) used in this study, i.e., dimethyl phosphate (X^1–0^), dibutyl phosphate (X^1–1^), didecyl phosphate (X^1–2^), dihexadecyl phosphate (X^1–3^), heptanoate (X^2–1^), undecanoate (X^2–2^), and heptadecanoate (X^2–3^) ligands.

Through the rational selection of the organic proligands, we extended our systematic studies on the influence of the nature of the ligand shell, in particular the type of anchored headgroup and the length of the ligand tail, on the properties of OSSOM-derived ZnO NCs’ self-assembly (Figure 1). The OSSOM approach was used as a model platform to synthesize a series of ligand-coated ZnO NCs.^32,35,37–40,49^ Commercially available organophosphates (X^1^-H) and respective carboxylic acids (X^2^-H) were used as proligands, namely, dimethyl phosphate (X^1–0^-H), dibutyl phosphate (X^1–1^-H), didecyl phosphate (X^1–2^-H), dihexadecyl phosphate (X^1–3^-H), heptanoic acid (X^2–1^-H), undecanoic acid (X^2–2^-H), and heptadecanoic acid (X^2–3^-H), for the preparation of a series of colloidal ZnO NCs. In general, organophosphate ligands are considered phosphorus-based analogs of carboxylate ligands; i.e., carboxylate and diorganophosphate anions are bidentate ligands, showing versatility in terms of binding modes. However, the monoanionic (RO)_2_PO_2_^–^ ligand features a tetrahedral geometry with two R-tail (where R = alkyl or aryl group and its combinations), which results in some differences in both the surface-binding modes relative to the planar RCO_2_^–^ carboxylate ligands as well as the rigidity and the density of the resulting ligand shell.^50^ Furthermore, diorganophosphates exhibit stronger coordination to metal centers compared to carboxylate ligands.^51^

The resulting ZnO-X NCs were characterized by employing scanning transmission electron microscopy (STEM), powder X-ray diffraction (PXRD), dynamic light scattering (DLS) measurements, thermogravimetric analysis (TGA), Fourier-transform infrared spectroscopy (FTIR), UV–vis spectrophotometry, and photoluminescence (PL) spectroscopy. All of the data concerning both intrinsic and extrinsic properties of the resulting phosphate-coated ZnO NCs are summarized in the Supporting Information (Table S1). The representative STEM micrographs of ZnO-X^1^ NCs are presented in Figure 2 and show well-dispersed and quasi-spherically shaped nanostructures. Nanocrystal size distributions calculated from high-resolution transmission electron microscopy (HRTEM) images revealed diameters of 4.1 ± 0.5, 5.1 ± 0.5, 4.8 ± 0.5, and 5.2 ± 0.9 nm for ZnO-X^1–0^ NCs, ZnO-X^1–1^ NCs, ZnO-X^1–2^ NCs, and ZnO-X^1–3^ NCs, respectively (Table S1 and Figure 2, right). The phase purity and degree of crystallinity were confirmed by PXRD measurements (Figure S1). The observed diffraction patterns corresponded well to standard wurtzite-type ZnO. The solvodynamic diameters (Z-average) of the resulting phosphate-ZnO NCs dispersed in THF were 12.2, 8.1, 9.1, and 9.1 nm for ZnO-X^1–0^ NCs, ZnO-X^1–1^ NCs, ZnO-X^1–2^ NCs, and ZnO-X^1–3^, respectively (Table S1). The FTIR spectra exhibit a sharp band in the 450–500 cm^–1^ range corresponding to the stretching Zn–O bond. The lack of characteristic absorption at ∼3600 cm^–1^ and the 2700–2500 cm^–1^ region confirms the monoanionic form of the ligand attached to the surface. The P–O–Zn stretching oscillations correspond to high-intensity bands at 1182 cm^–1^ and 1021 cm^–1^. Notably, a broad and diffuse band in the range 3500–3000 cm^–1^ corresponds to the presence of residual water molecules (Figure S2). Furthermore, in the TGA profiles of ZnO-X^1–1^ NCs and ZnO-X^1–2^ NCs, single primary decomposition steps at 283 and 293 °C with total weight losses of 42 and 55 wt %, respectively, were observed (Figure S3). The thermolysis of ZnO-X^1–0^ and ZnO-X^1–3^ showed a slightly more complex decomposition path involving two main steps at 256 °C, 448 °C (37 wt %), and 230 °C, 273 °C (34 wt %), respectively. The NCs revealed a broad absorption in the ultraviolet region of 335–345 nm and broad PL emission peaks centered between 530 and 555 nm (Table S1 and Figure S4). All ZnO NCs stabilized with respective carboxylate-type ligands exhibit physicochemical properties similar to previously described systems^31,34–40^ and are summarized in the Supporting Information (Table S2, Figures S5–S7).

**Figure 2:**
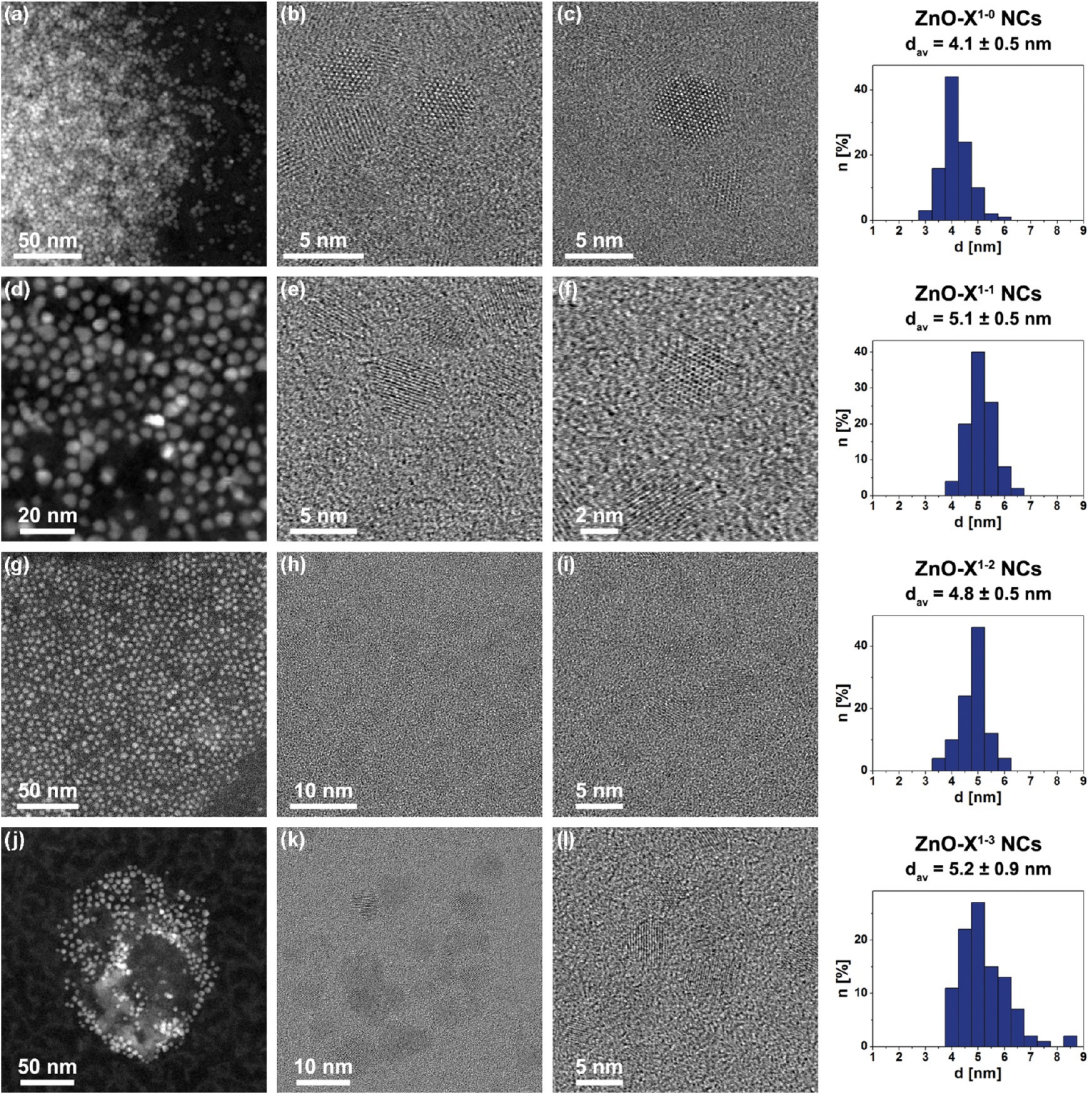
Representative STEM images of (a–c) ZnO-X^1–0^, (d–f) ZnO-X^1–1^, (g–i) ZnO-X^1–2^, and (j–l) ZnO-X^1–3^ NCs in SE, and HRTEM modes along with their size distributions.

### OSSOM-Derived NC’s Self-Assembly at the Air–Water Interface

Our recent works demonstrated the self-assembly of carboxylate-coated ZnO NCs within thin films.^34,36^ We showed that anionic X-type carboxylate ligands with long-chain functionality, including liquid crystalline-type ligands, allowed for the interdigitation of ZnO NCs, which resulted in free-standing and freely suspended thin films.^36^ We also showed that liquid crystalline ligands induced order within photoactive superlattices of ZnO NCs.^34^ Thus, in this work, we attempted to determine the impact of the character of the ligand shell on the self-assembly properties of the ZnO NCs. The change in the character of a coating ligand, particularly the type of anchored headgroup and the length of the ligand alkyl tail, may result in a more dense organic shell, which limits the interdigitation process as well as ligand–ligand interactions. We have already shown that introducing bulky-type ligands (i.e., polyoctahedral silsesquioxanes, POSS) affected the self-assembly process at the air–water interface^52^ by preventing the ligand–ligand interdigitation between the neighboring NCs.

First, we established the dependence between the spread volume of the studied ZnO NCs suspensions and the contact cross-sectional area (CCSA) (Figure 3a). CCSA values were determined by drawing tangents to the part of the π–A isotherms where the surface pressure increases. We found that ZnO NCs coated with dimethyl phosphate ligands (ZnO-X^1–0^) did not form stable films at the air–water interface (Figure S8). Short tail-type dimethyl phosphate ligands did not provide enough hydrophobicity to support the particles at the water surface and form stable films upon compression. For ZnO-X^1–1^, ZnO-X^1–2^, and ZnO-X^1–3^, the relationship between CCSA and the spreading volume was approximately linear (see inset in Figure 3a). The constant spreading ability proved that films were uniform and no or minimal 3D aggregates were present at the air–water interface during the first compression.^53^ Consecutive compression–decompression cycles were shifted toward smaller surface area values, suggesting that particles desorbed from the interface (either toward the air or into the subphase) (Figure S9).

**Figure 3:**
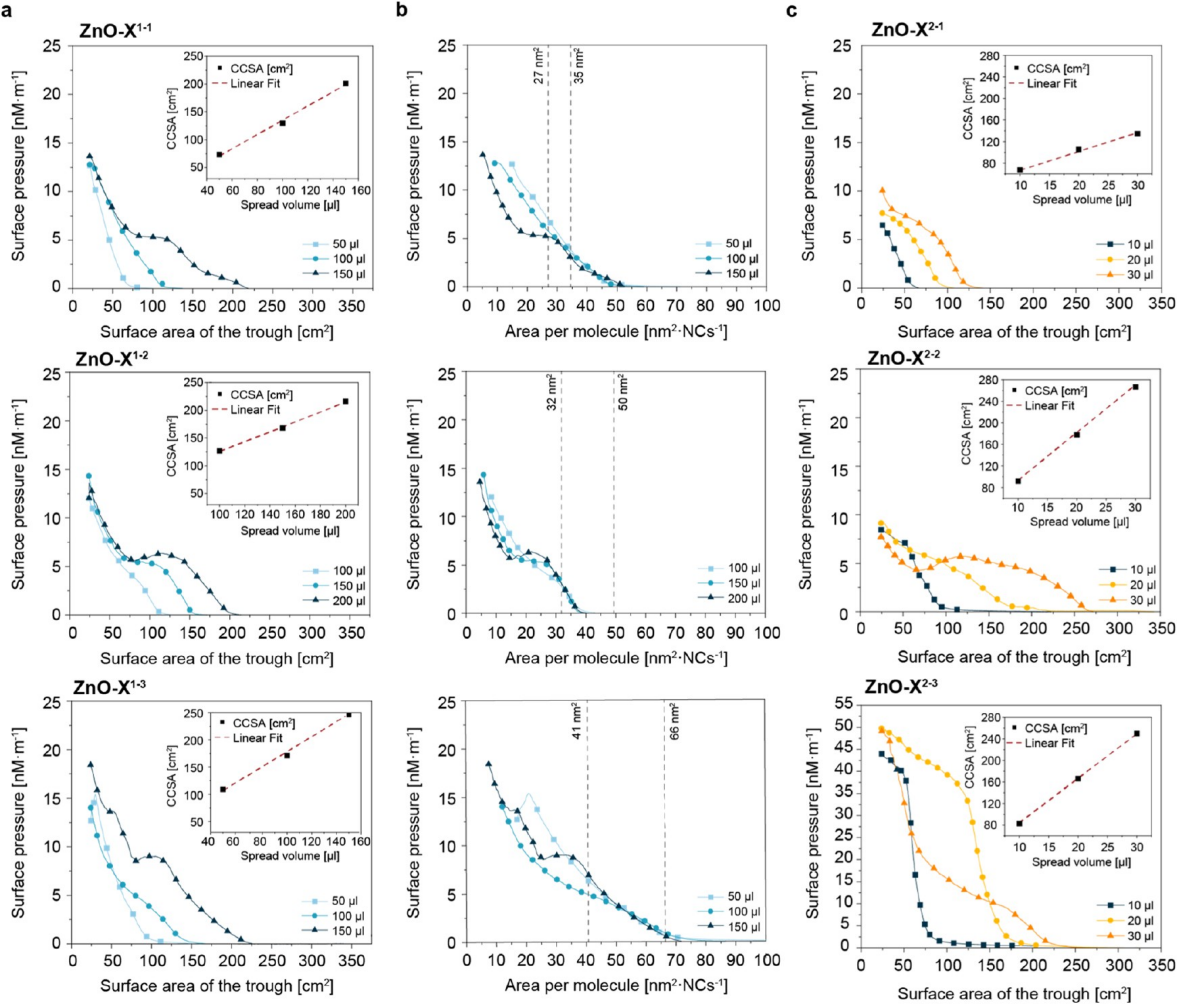
Surface pressure versus area isotherms of ZnO NCs coated with (a) phosphate (ZnO-X^1–1^, ZnO-X^1–2^, ZnO-X^1–3^) and (c) carboxylate ligands (ZnO-X^2–1^, ZnO-X^2–2^, ZnO-X^2–3^) with varying tail lengths recorded for various volumes of suspensions applied on the air–water interface. Insets show contact cross-sectional area (CCSA), which increased linearly with increased volumes of NCs suspensions applied onto the air–water interface; (b) compression isotherms expressed as a function of area per single phosphate-coated ZnO NCs.

To verify the influence of the type of anchored headgroup and the density of aliphatic chains on the films′ properties, we decided to use carboxylate ligands as analogs of organophosphate ligands. Representative isotherms for the respective carboxylate-coated ZnO NCs can be found in Figure 3c. ZnO-X^2–1^ NCs did not form stable films at the air–water interface (an increase in volume spread by a factor of 3 increased CCSA only by around two times). The planar geometry of carboxylate groups (RCO_2_®) may have contributed to the observed instability, as the alkyl tails of these ligands might not provide the necessary interdigitation and close packing required to form a robust monolayer. However, intriguingly, when examining phosphate-coated NCs with the same length of alkyl ligand tail (ZnO-X^1–1^), we observed the formation of stable films (Figure 3a). The tetrahedral geometry of phosphate head groups ((RO)_2_PO_2_®) likely facilitated improved NC surface coverage, promoting stable monolayer formation. This contrast underscored the pivotal role of ligand headgroup geometry in influencing the stability of nanocrystal-based thin layers. Notably, our investigations revealed that extending the length of the ligand alkyl tail of carboxylate-coated ZnO NCs improved the stability of monolayer formation for ZnO X^2–2^ and ZnO-X^2–3^ NCs, respectively.

From compression isotherms, the surface compressional modulus was inferred according to the equation $\varepsilon_{S}=-A{\left(\frac{\partial \pi }{\partial A}\right)}_{T}$ .^54^ *ε*_s_ is the reciprocal of compressibility *C*_s_. By analyzing the elasticity, it is possible to understand the state of the monolayer and its changes, i.e., molecular arrangement, phase structure, and phase transition. The maximum values of *ε*_s_ observed for phosphate-coated ZnO NCs were in the range from below 15 mN·m^–1^ (for ZnO-X^1–1^ and ZnO-X^1–3^) to above 20 mN·m^–1^ (ZnO-X^1–2^). Surprisingly, the highest elasticity was observed for the middle length ligand. Elasticity values corresponded to the gaseous (for ZnO-X^1–1^ and ZnO-X^1–3^) and liquid-expanded (ZnO-X^1–2^) phases according to classical work by Davies and Rideal.^55^ Interestingly, the maximal values of surface elasticity were observed at around 30 nm^2^·NC^–1^ for ZnO-X^1–1^ and ZnO-X^1–2^ and 60 nm^2^·NC^–1^ in the case of ZnO-X^1–3^, i.e., the tendencies were different for maximal values and areas at which these values appeared (Figure S10). This indicated different interactions between ZnO-X^1–3^ compared to ZnO-X^1–1^ and ZnO-X^1–2^.

We recalculated the obtained results to show the surface pressure changes expressed in nm^2^ per nanocrystal (nm^2^·NC^–1^) analogously to molecular amphiphiles. In such a case, isotherms recorded for various amounts of suspensions deposited onto the air–water interface overlapped almost perfectly (Figure 3b). We marked (as dashed lines) the calculated values of area per nanoparticle assuming both full interdigitation (distance between neighboring NCs equal to the length of a single ligand, i.e., $A=\pi {\left(\frac{D}{2}+\frac{L}{2}\right)}^{2}$ , where *D* is the diameter of NCs and *L* is the length of the ligand) and no interdigitation (distance between neighboring NCs equal to 2× length of a single ligand, i.e., $A=\pi {\left(\frac{D}{2}+L\right)}^{2}$ ). Lengths of ligands were assumed based on the CPK model.^46,47^ The diameters of the cores were inferred from electron microscopy measurements. For ZnO-X^1–1^ and ZnO-X^1–2^, the observed values of the area per NCs at the inflection of the isotherm (presumably collapse) were close to calculated values for full interdigitation (i.e., around 27 and 31 nm^2^·NC^–1^, respectively). This was also in line with areas of maximal values of surface elasticity (Figure S10), which for these NCs were around 30 nm^2^·NC^–1^. However, in the case of ZnO-X^1–3^, there was no clear inflection point at the isotherm, and the maximal surface compressional modulus was observed at 60 nm^2^·NC^–1^. This was close to the estimated value of the surface area for no interdigitation (66 nm^2^·NC^–1^).

Lift-off areas recorded for ZnO NCs coated with short ligands (ZnO-X^1–1^; around 40 nm^2^·NC^–1^) were larger than those for ZnO NCs coated with medium ligands (ZnO-X^1–2^; around 35 nm^2^·NC^–1^). This apparent discrepancy can be explained by analyzing ligand-mediated interactions between ZnO NCs. Interactions between shorter ligands (ZnO-X^1–1^) were weaker, and films had a less dense structure, resulting in a higher lift-off area value. In contrast, longer stabilizing ligand chains interacted due to van der Waals or hydrophobic interactions. Dihexadecyl phosphate ligands (long-type ligand) capping ZnO-X^1–3^ were long enough, so the lift-off area was, as expected, the largest among the studied nanocrystals (around 70 nm^2^·NC^–1^) even despite strong interparticle interactions. Similar results were obtained by us previously in the study on amphiphilic hyperbranched polyesters with end groups modified with hydrophobic chains of various lengths.^56^

The compression isotherms of the nanocrystal films displayed features sensitive to both the amount of material deposited and the dynamics of film compression. In particular, the presence of a pronounced inflection in the isotherms (e.g., ZnO-X^2–3^) at higher deposition volumes was attributed to the onset of monolayer collapse or structural reorganization, such as buckling or wrinkling, as the film exceeds its packing limit. Additionally, kinetic factors played an important role: a greater surface density of nanocrystals at a constant barrier speed results in a higher effective compression rate per particle, leaving less time for local rearrangement. This led to out-of-equilibrium effects, such as jamming and clustering, reflected in irregular features of the isotherm. Any such effect was more probable when the area of the film was larger, i.e., at a higher volume of deposited NCs suspensions. Similar kinetic influences on isotherm shape have been observed previously for molecular films under lateral compression, as described by Kwok et al.^57^

Brewster angle microscopy (BAM) observations gave additional insight into the particle interactions. The structure of films composed of ZnO-X^1–1^ (short ligand) changed only slightly upon compression (Figure 4). The two-dimensional gas phase was dominant, confirming the analysis of *ε*_s_. Compression beyond the inflection point (possible collapse) did not change the film’s structure, and the density of objects at the interface did not change significantly. This suggested that instead of 3D aggregation, the injection of particles into the subphase occurred. In the case of longer stabilizing ligands (ZnO-X^1–2^ and ZnO-X^1–3^), particles interacted spontaneously even before compression, causing the formation of larger domains during the equilibration period. This indicated stronger interparticle interactions caused solely by longer ligands (which confirmed the explanation of lift-off areas). Upon compression, the domains were merged. A denser phase was visible in both cases compared to short-type ligand-coated ZnO-X^1–1^ NCs.

**Figure 4:**
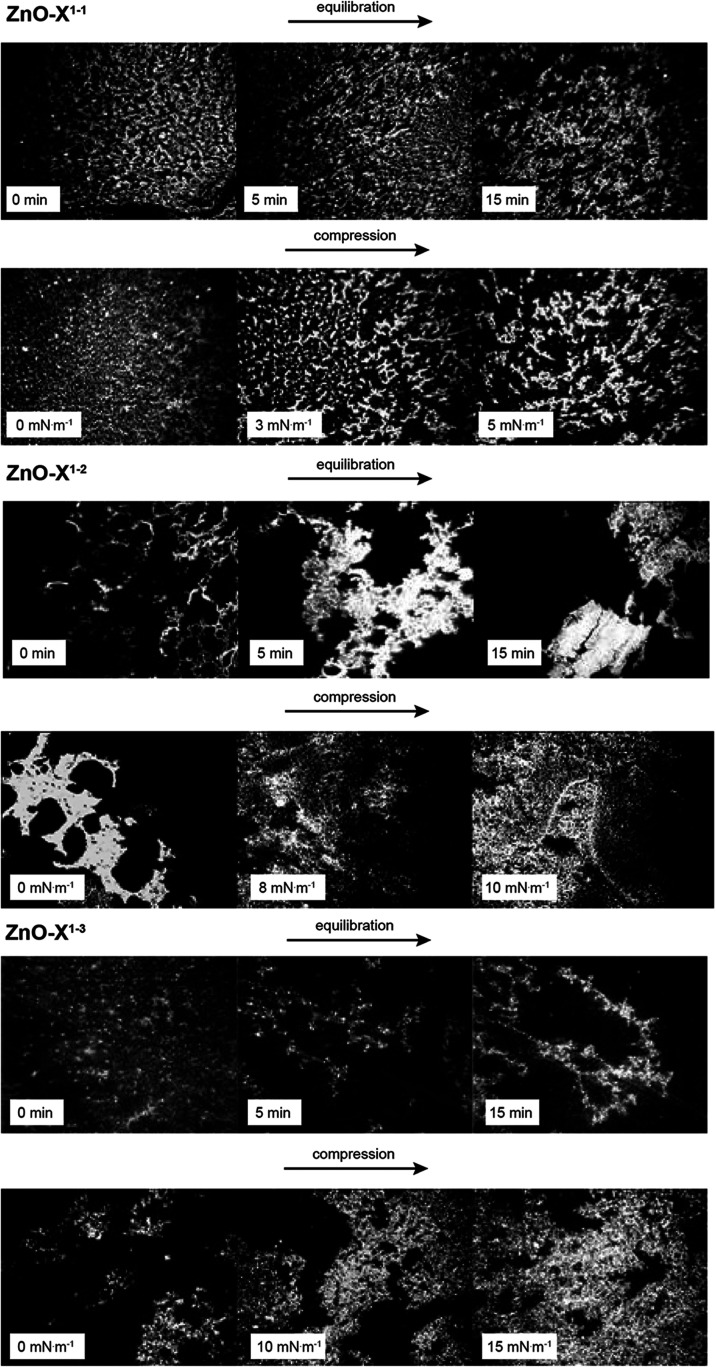
BAM images showing the morphology of the studied phosphate ligand-coated ZnO NCs before and during compression. The size of the field of view in the BAM images is 6.4 × 4.8 mm.

Finally, we examined the viscoelastic properties of the monolayers composed of ZnO NCs (Figure 5). This allowed for a more accurate analysis of the interactions among particles coated with short-, medium-, and long-type organic ligands. We studied the films’ dynamic behavior through the oscillating barriers method. The surface dilatational elasticity modulus (*E*) is derived from the impact of small changes in surface area (dilatational strain) on surface pressure (dilatational stress) according to the relation $E={-\left(\frac{\partial \pi }{\partial \;\ln A}\right)}_{T}$ .^58,59^ The surface dilatational elasticity modulus comprises the real component *E*_d_ (interfacial dilatational elasticity modulus) and the imaginary component *E*_v_ (interfacial dilatational viscosity modulus). Therefore, *E*_d_ = |*E*| cos *θ* and *E*_v_ = |*E*| sin *θ*. *θ* is a phase angle, i.e., the delay between dilatational stress and dilatational strain. For a purely elastic material, *θ* = 0°, whereas for a purely viscous material, *θ* = 90°.^60^ We recorded the dynamic response at low surface pressures, between 1.5 and 5 mN·m^–1^, close to maximal values of surface compressional moduli, to ensure the linearity of the response. The mechanical response observed at low surface pressures, where the monolayers remain in a relatively dilute and dynamic state, reflects ligand–ligand interactions, such as steric repulsion, van der Waals attraction, and potential interdigitation. Even if some level of aggregation was present, especially for the longest ligands, these partially disordered assemblies still encoded ligand-specific effects that govern the compressibility and viscoelastic properties of the films.

**Figure 5:**
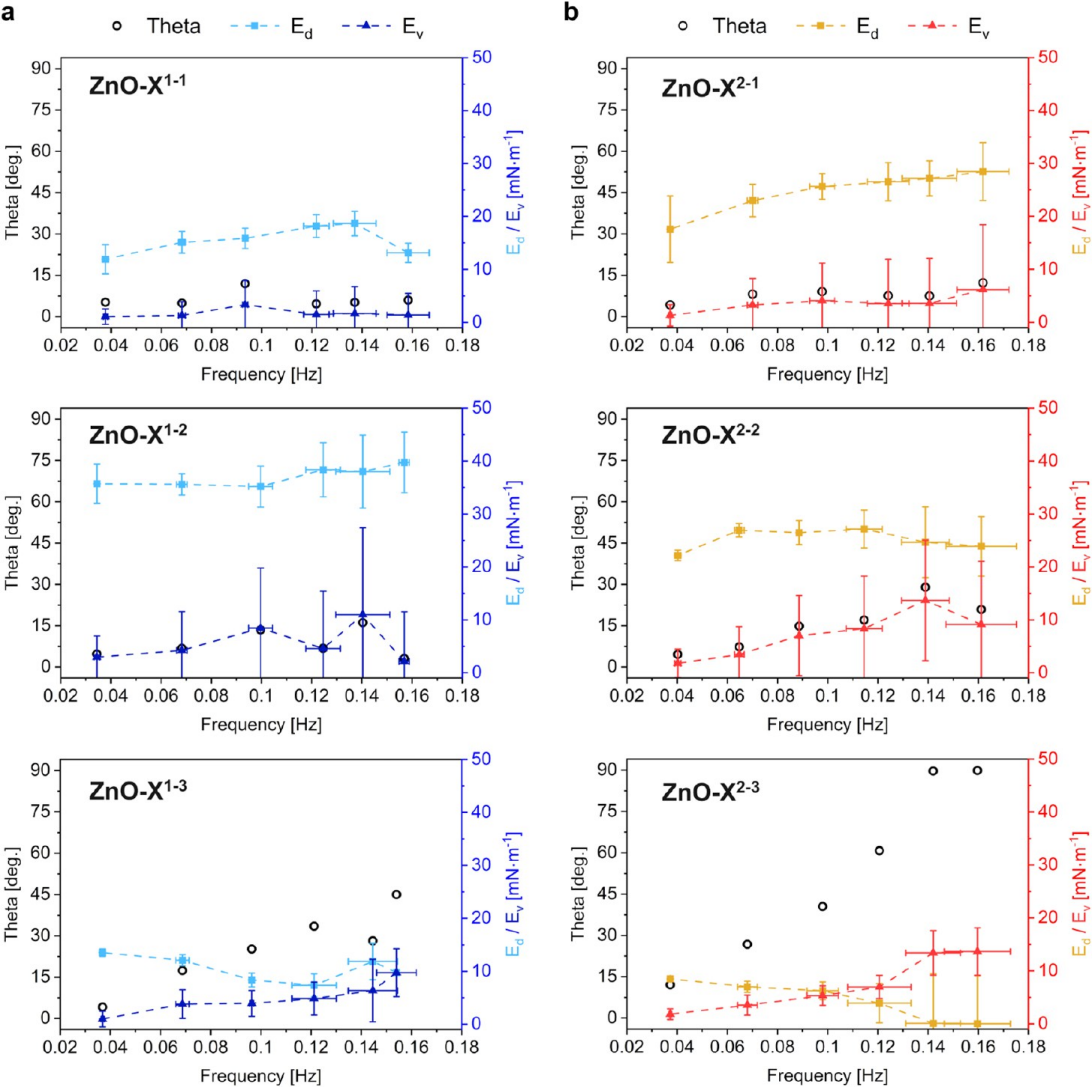
Viscoelastic properties of monolayers under dilatational deformations: (a) phosphate ligand-coated NCs (ZnO-X^1–1^ NCs – ZnO-X^1–3^) and (b) carboxylate ligand-coated NCs (ZnO-X^2–1^ NCs – ZnO-X^2–3^).

We compared phosphates, which form more dense organic ligand shells (two alkyl chains per ligand), to carboxylates, whose linear structure (one alkyl chain per ligand) facilitates the interdigitation of surface ligands of neighboring particles. When ligands were short (ZnO-X^1–1^ and ZnO-X^2–1^), the character of the films of both phosphate and carboxylate-coated ZnO NCs was dominated by the elastic component, i.e., *θ* was below 10° in the whole range of studied frequencies. ZnO-X^2–2^ (coated with medium-type carboxylate ligands) started showing viscoelastic properties, with the *θ* angle reaching around 30°. For the medium phosphate ligand (ZnO-X^1–2^), *θ* still did not exceed 15°. The difference between phosphate and carboxylate ligands was even more pronounced for the longest ligands. *θ* reached 90° for the carboxylate ligand (ZnO-X^2–3^), which showed a very viscous character of the films and correlated to aliphatic chains interacting with ligand coatings of neighboring NCs. In the case of phosphate ligand (ZnO-X^1–3^), the maximum *θ* value was around 45°; i.e., the films had a more elastic character, especially in comparison with the carboxylate-protected analog. *θ* value around 45° corresponding to tan *θ* = 1. The loss angle tangent is defined by the ratio between the imaginary and real components of the dilatational modulus $\tan\theta =\frac{E_{v}}{E_{d}}$ . This is also a threshold that differentiates films of more viscosity from those with more elastic properties.

The change in the character of the organic ligand shell, particularly the type of anchored headgroup and the length of the ligand tail group, was analyzed. For longer ligands, there was more space between chains available for interdigitation due to the curvature of the NCs. Therefore, a more viscous character was observed for long versus medium and short ligands in phosphate- and carboxylate-coated NCs.

The difference in *θ* observed between carboxylates and phosphate for medium (ZnO-X^2–2^ and ZnO-X^1–2^) and long (ZnO-X^2–3^ and ZnO-X^1–3^) ligands underlined the importance of the density of the organic shell. A dense organic shell (two aliphatic chains per ligand in phosphates compared to single aliphatic chains in the case of carboxylates) hinders interdigitation even for long ligands, as the difference between ZnO-X^2–3^ and ZnO-X^1–3^ proved.

*E*_d_ became larger than *E*_v_ for ZnO-X^2–3^ as frequency increased, i.e., the transition from the elastic to the viscous character of the film. This was due to strong interactions between particles, which kept particles together on time scales longer than allowed by high-frequency oscillations. This was the only case among the studied compounds as two conditions were met: (i) the ligands were long enough, and (ii) there was enough space between ligands for capping layers to penetrate each other (and thus, this was not visible in ZnO-X^1–3^).

There was an additional noticeable difference between films of carboxylate-coated ZnO NCs and films of phosphate-coated ZnO NCs. Upon compression, films composed of ZnO-X^2–2^ and ZnO-X^2–3^ became turbid.^36^ Our previous work showed that this was due to the interdigitation of the ligands. This allowed for the creation of freely suspended and free-standing films of thicknesses of up to a few hundred nanometers. Such structures were photoactive as ligands protected NCs against coalescence.^36^ In the case of phosphate-coated films, we observed the turbidity of the films only for ZnO-X^1–3^ and at very high surface pressures. This simple observation was consistent with the experimental techniques used in the study. Moreover, the solvodynamic diameter of ZnO-X^2–3^ was much larger compared to other studied systems (Tables S1 and S2), likely due to some level of aggregation, supporting the pronounced ligand interdigitation in this case.

### Langmuir–Blodgett Films of Phosphate-Coated ZnO NCs

To gain better insight into the structure and morphology of the thin films, we transferred them onto solid, hydrophilic substrates according to the Langmuir–Blodgett method. Then, the resulting films were analyzed by employing scanning electron microscopy (SEM) and X-ray reflectivity measurements (XRR) (Figure 6). The SEM images revealed apparent differences in the morphology of thin films composed of phosphate-coated ZnO NCs depending on the length of the alkyl tail of the ligand (Figure 6c). In the case of the shorter alkyl tail (ZnO-X^1–1^), nanocrystallites were relatively sparsely distributed and formed chain-like quasi-fractal open structures. Shorter alkyl chains might lead to weaker interactions between particles due to reduced steric hindrance and less surface area available for interactions. As a result, the self-organization might be less pronounced, and the particles were more prone to remaining dispersed or forming loosely connected structures. Such structures were previously found in mixtures of nanoparticles and amphiphilic^61^ or polymeric additives.^62^ It was also shown that single-component systems might not cluster together or relax into the homogeneous bulk phase but form highly porous structures in the out-of-equilibrium states.^63^ As the length of the alkyl tail in the ligand increased (ZnO-X^1–2^), nanocrystallites assembled into more tightly packed structures, affected by attractive ligand–ligand interactions (van der Waals forces) and the interdigitation of ligands between neighboring NPs. Further elongation of the alkyl tail of the ligand (ZnO-X^1–3^) contributed to stronger interactions, enabling the formation of rod-like aggregates of micrometer size characterized by anisotropic shapes, which were likely responsible for the turbidity of films. Overall, this illustrates how the degree of self-organization varies with the length of the alkyl chains coating the particles.

**Figure 6:**
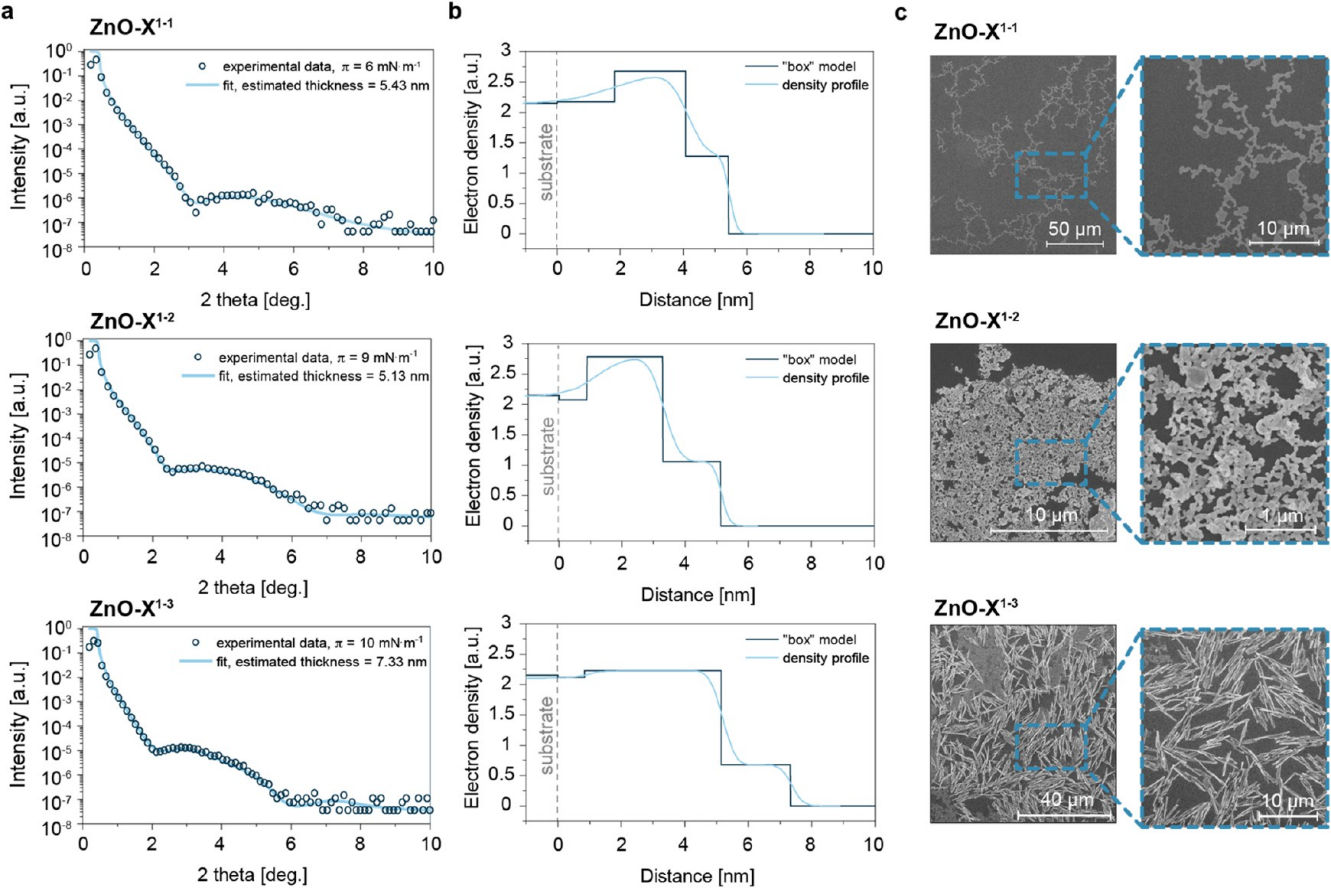
(a) X-ray reflectivity patterns and simulated fit curve for phosphate ligand-coated ZnO NCs (ZnO-X^1–1^–ZnO-X^1–3^) thin films transferred to silicon wafers at surface pressures of 6, 9, and 10 mN·m^–1^, according to the Langmuir–Blodgett method; (b) density profile used for XRR pattern simulation; (c) SEM images of ZnO NCs thin films deposited on solid substrate.

XRR patterns showed thickness from around 5 to 7 nm, which was in line with the size of a single nanocrystallite (Figure 6a). This suggested that recorded XRR patterns corresponded to monolayers of ZnO NCs. Also, the density profiles used for the fitting agreed with the expected structure of the films (Figure 6b). This result was somehow surprising in the case of ZnO-X^1–3^, where aggregates were visible under SEM. At least two explanations could rationalize these seemingly contradictory results. First, 3D aggregates did not occupy a significant surface area and thus did not contribute significantly to the XRR pattern. The fitted density of the cores sublayer within the ZnO-X^1–3^ film was lower than ZnO-X^1–1^ and ZnO-X^1–2^, proving lower surface coverage. Missing particles created aggregates above the monolayer film. This statement was also based on the analysis of compression isotherms. Second, it was also likely that these aggregates were not ordered, so there was no positive interference of X-rays to be detected. In our previous work, we showed that the ordering within the aggregates was introduced by liquid crystalline ligands^34^ but was not observed in the case of ligands with aliphatic tails (as in ZnO-X^1–3^).

The appearance of the aggregates also explained why the maximal surface compressional modulus for ZnO-X^1–3^ was smaller than that for ZnO-X^1–2^ (below 15 mN·m^–1^ versus above 20 mN·m^–1^) and was recorded for large values of surface area (60 nm^2^·NC^–1^ versus 30 nm^2^·NC^–1^). ZnO-X^1–3^ particles were more hydrophobic than ZnO-X^1–2^ and desorbed more easily from the air–water interface. This resulted in the formation of aggregates upon compression above a certain surface pressure, most likely above around 5 mN·m^–1^. Therefore, the aggregates are visible in Figure 6c, as the investigated films were transferred at around 10 mN·m^–1^.

Additionally, analysis using profilometry was executed, confirming SEM and XRR observations. These results are discussed in detail in the Supporting Information.

## Conclusions

We demonstrate how the character of a ligand shell, particularly the type of anchored headgroup and the length of the ligand alkyl tail, affects the self-assembly properties of ZnO NCs at the air–water interface and the properties of corresponding thin films. Based on the analysis of surface isotherms, BAM pictures, elastic properties (both dynamic and static), XRR, and SEM, we found that the density of the capping organic shell is a crucial, yet often underestimated, factor affecting the self-assembly properties of nano-objects. Nanocrystallites coated with phosphate ligands with short aliphatic chains behaved as elastic spheres; i.e., interparticle interactions are weak, resulting in 2D gas-like phase formation. The morphology of films on solid substrates corresponded to structures observed for weakly interacting particles described before.^63^ Increasing the length of the aliphatic chains of the stabilizing phosphate ligands resulted in the appearance of a liquid-like phase at the air–water interface. Islands composed of ZnO NCs were visible both using BAM directly at the air–water interface and within films transferred onto a solid substrate. Dynamic elasticity experiments showed only a small contribution of the viscoelastic component (*θ* did not exceed 15°). More viscoelastic properties were evident for the longest studied ligand, i.e., dihexadecyl phosphate-coated ZnO-X^1–3^ (*θ* = around 45°). In this case, 3D aggregates were formed due to the interdigitation of the ligands upon compression above the threshold surface pressure. The choice between phosphates (two aliphatic chains per ligand) and carboxylates (single aliphatic chain per ligand) affected the organic layer’s density and rigidity. Capping shells of carboxylate-protected ZnO NCs interdigitated significantly, as there was more space for ligands to interpenetrate the organic shells of neighboring particles. This resulted in *θ* of around 90° for ZnO-X^2–3^, i.e., nanocrystals coated with heptadecanoate ligands showed viscous dominance over elastic dominance in the character of the film. To substantiate that the rheological properties reflect ligand–ligand interactions rather than irreversible aggregation or core fusion, we note that ZnO NCs retain their photoactivity even when assembled into large-scale superstructures, indicating that the NC cores remain structurally stable across a range of assembly states.^34^ Moreover, previous studies demonstrated that bulky surface ligands effectively prevent interdigitation and strongly influence interfacial packing behavior.^52^ These findings are consistent with the ligand-dependent rheological responses observed in our current study and support the interpretation that interfacial mechanical properties are governed by molecular-scale ligand architecture, even in partially ordered or disordered assemblies.

Our findings on ZnO NCs presented here show new aspects compared to our previous works.^34,36,52^ This difference seems to be due to the diverse nature of the bond between the core and the ligand. Rigidity caused by the well-defined ligand–ZnO bond allows ligands bonded to the oxide surface to interpenetrate. The capping layer flexes without interdigitation when ligands are labile (as in the case of a metal–thiol bond). It was proved in the past that an increase in molecular stiffness allows for the formation of complex, multilayered systems of bolaamphiphiles at the air–water interface.^64–66^ In the case of nanoparticulate systems, control over the parameters of organic shells gives another possibility to tune the properties of desired self-assembly systems, including the potential to form superlattices or NC solids.

The ability to precisely control the lateral organization of nanocrystals at interfaces is not only fundamentally intriguing but also pivotal for various emerging technologies. In particular, highly ordered nanocrystal monolayers are key to optimizing charge transport in optoelectronic devices such as quantum-dot-based light-emitting diodes (QLEDs), field-effect transistors (FETs), and photodetectors. Moreover, in applications such as photocatalysis and chemical sensing, the packing density and spatial arrangement of NCs can govern surface accessibility and collective optical or electronic phenomena. Thus, understanding how surface chemistry guides interfacial assembly provides essential design principles for engineering functional nanomaterials.

## Supplementary Material



## Notes

Detailed characterization of studied ZnO NCs and additional experimental results after compression of ZnO NCs at the air–water interface (PDF)
