# Supplementary material for: Unraveling the Influence of the Anchored Headgroup and Ligand Tail Group Length on the Self-Assembly of ZnO NCs at the Air–Water Interface
Source: ACS Appl Mater Interfaces. 2025 Jun 10;17(24):36212–25. doi: 10.1021/acsami.5c07324 (PMC12186221; doi:10.1021/acsami.5c07324)
Supplement: Supplementary file 1 [file am5c07324_si_001.pdf]

Supporting Information

Unraveling the influence of the anchored head group and ligand tail group length on self-assembly of ZnO NCs at the air-water interface

Aleksandra Borkenhagen,[a] Małgorzata Wolska-Pietkiewicz,[,b] Ilona Binkiewicz,[a,b]*

Łukasz Richter,[a] Rafał Zbonikowski,[a] Jan Paczesny,[,a] and Janusz Lewiński[*,a,b]*

[a] Institute of Physical Chemistry PAS,

Kasprzaka 44/52, 01-224 Warsaw, Poland.

[b] Faculty of Chemistry, Warsaw University of Technology,

Noakowskiego 3, 00-664 Warsaw, Poland.

Corresponding authors:

* Małgorzata Wolska-Pietkiewicz, email Malgorzata.Pietkiewicz@pw.edu.pl

* Jan Paczesny, email: jpaczesny@ichf.edu.pl

* Janusz Lewiński, email: jlewinski@ichf.edu.pl

Table of contents

Phosphate-coated ZnO NCs 3

PXRD analysis 4

FTIR analysis 5

Thermogravimetric analysis 6

Steady-state spectroscopy 7

Reference carboxylate-coated ZnO NCs 7

PXRD analysis 8

Thermogravimetric analysis 9

Steady-state spectroscopy 10

OSSOM-derived NC's self-assembly at the air-water interface 11

Langmuir films of ZnO-X^{1-0} NCs 11

Reproducibility of Langmuir isotherms upon compression-decompression cycles 12

Surface compressional modulus of Langmuir monolayers 12

Films transferred onto the solid substrates 13

Profilometry analysis 13

Phosphate-coated ZnO NCs

Table S1. Physical characterization data for phosphate-coated ZnO NCs.

	Core size [nm]	SD [nm]	PdI	λ_{abs} [nm]	λ_{em} [nm]
**ZnO-X$^{1-0}$**	4.1 ± 0.5[a]	12.2$^{Z\text{-}av}$	0.22	345	540
**ZnO-X$^{1-1}$**	5.1 ± 0.5[a]<br>4.05[b]	8.1 $^{Z\text{-}av}$	0.09	335	530
**ZnO-X$^{1-2}$**	4.8 ± 0.5[a]<br>5.09[b]	9.1$^{Z\text{-}av}$	0.22	337	530
**ZnO-X$^{1-3}$**	5.2 ± 0.9[a]<br>4.93[b]	9.1$^{Z\text{-}av}$	0.04	335	555

Abbreviations: core size estimated from [a]- TEM measurements, [b]- PXRD measurements; SD – solvodynamic diameter is given as Z-average size ($^{Z\text{-}av}$) value; PDI - polydispersity index; λ_{abs} – absorbance maximum wavelength; λ_{em} – emission maximum wavelength.

PXRD analysis

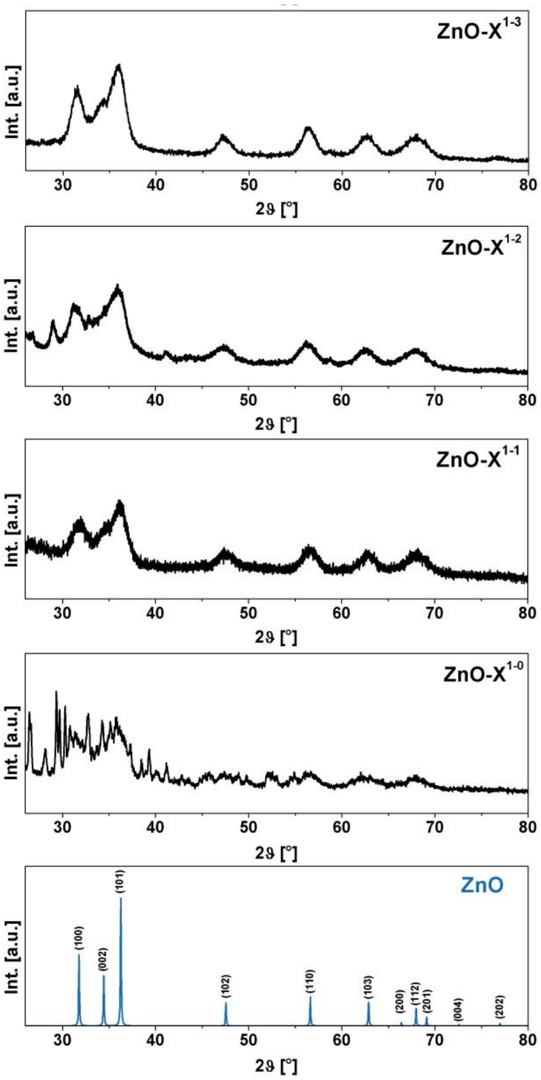

Figure S1. Powder X-ray diffraction pattern of ZnO-X$^{1-0}$ – ZnO-X$^{1-3}$ NCs in comparison with bulk ZnO pattern (blue line) confirming wurtzite crystalline core structure (note that no other ZnO-related crystalline phases are detected; the additional signals observed below 30° 2θ are most likely attributed to zinc phosphate species).

Note: ZnO NCs' core sizes were calculated from the Scherrer's formula:

$$d = \frac{k \cdot \lambda}{\beta \cdot \cos\theta}$$

where:
d - diameter of the inorganic core;
k - Scherrer's constant (crystallite-shape factor), **k = 0.89**;
λ - wavelength of the X-rays, **λ = 1.54 Å**;
θ - Bragg diffraction angle;
β - full-width at half-maximum of the X-ray diffraction peak.

FTIR analysis

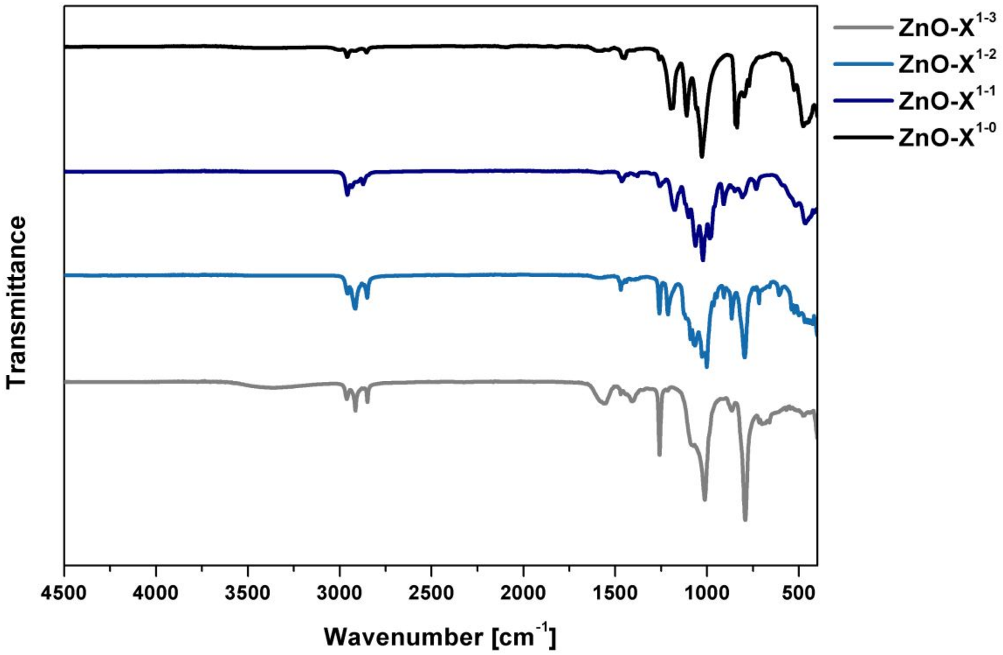

Figure S2. FTIR spectra of ZnO-X$^{1-0}$ - ZnO-X$^{1-3}$ NCs.

Thermogravimetric analysis

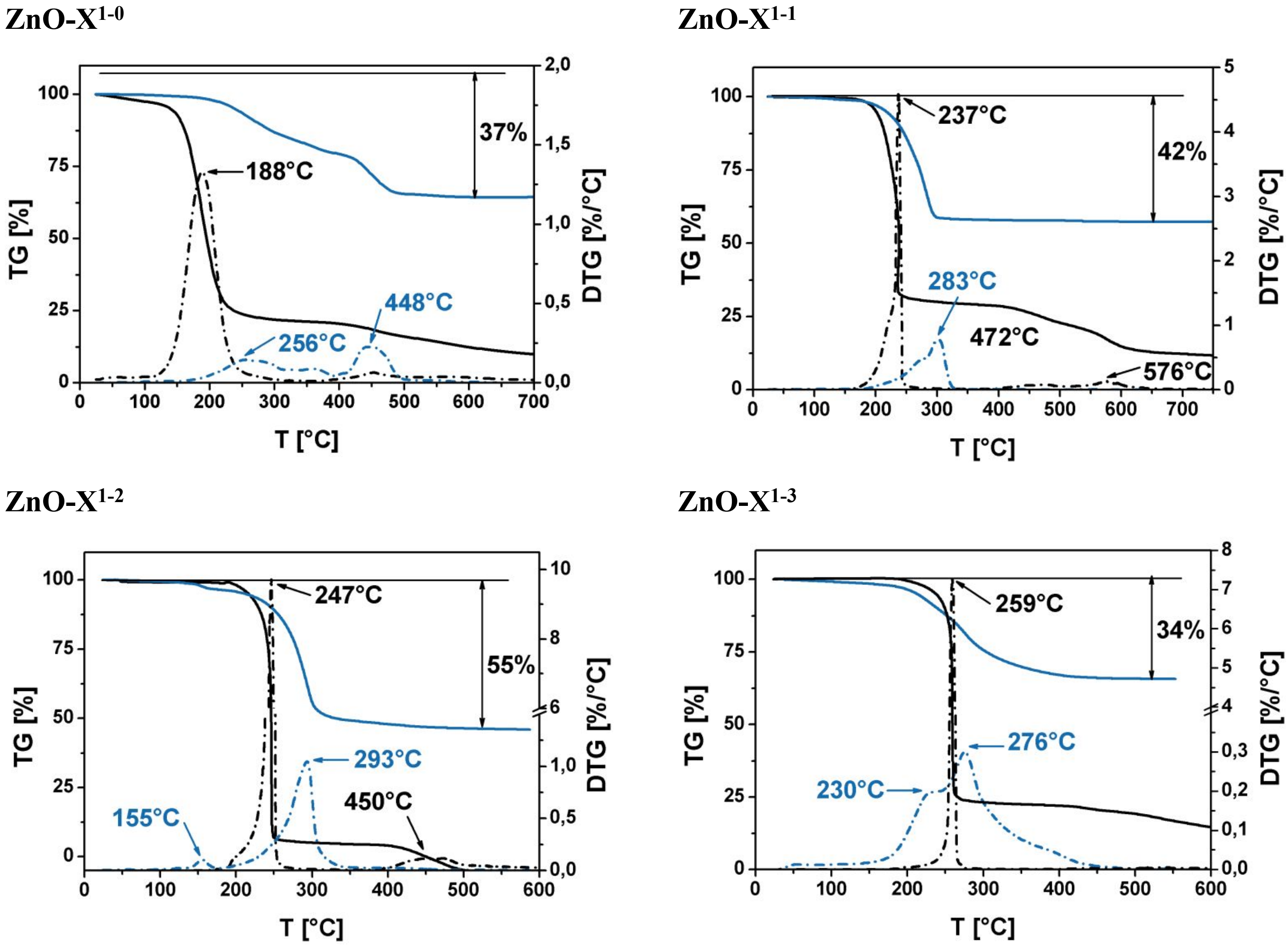

Figure S3. The TGA and derivative thermogravimetric analysis (DTG) traces showing the decomposition of ZnO-X^{1-0} - ZnO-X^{1-3} NCs (blue line) and the corresponding diorganophosphate proligands (black line) in an air atmosphere.

Steady-state spectroscopy

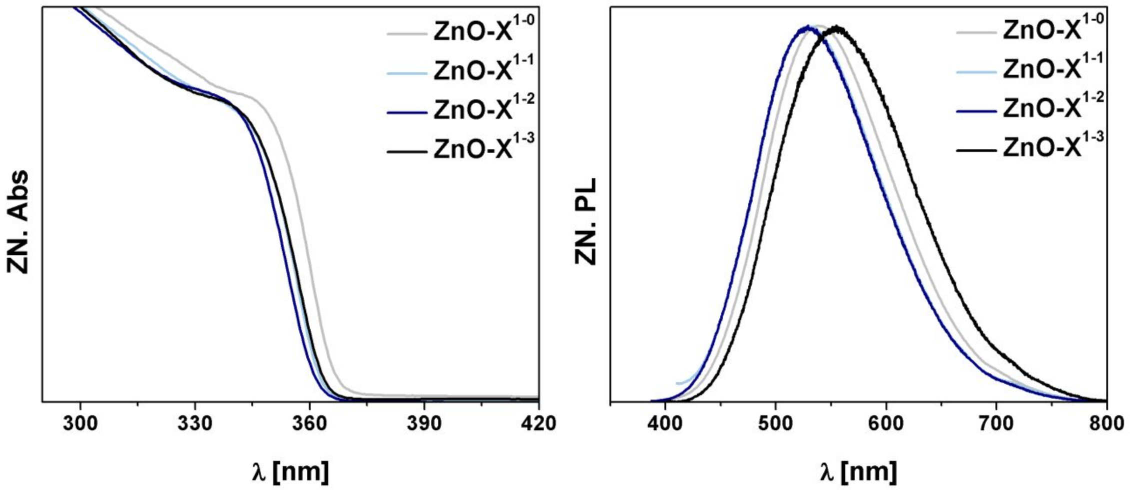

Figure S4. Normalized absorption (left) and emission (right) spectra of as-prepared diorganophosphate-coated ZnO NCs collected in THF.

Reference carboxylate-coated ZnO NCs

Table S2. Physical characterization data for reference carboxylate-coated ZnO NCs.

	Core size [nm]	SD [nm]	PdI	λ_{abs} [nm]	λ_{em} [nm]
**ZnO-X$^{2-1}$**	3.7 ± 0.8	8.64$^{Z\text{-}av}$	0.03	335	528
**ZnO-X$^{2-2}$**	3.3 ± 0.7	8.20$^{Z\text{-}av}$	0.02	345	530
**ZnO-X$^{2-3}$**	3.9 ± 0.7	58.62$^{Z\text{-}av}$	0.08	340	532

Abbreviations: core sizes were estimated from PXRD measurements; SD – solvodynamic diameter is given as intensity-averaged ($^{int.}$) values; PDI - polydispersity index; λ_{abs} – absorbance maximum wavelength; λ_{em} – emission maximum wavelength.

PXRD analysis

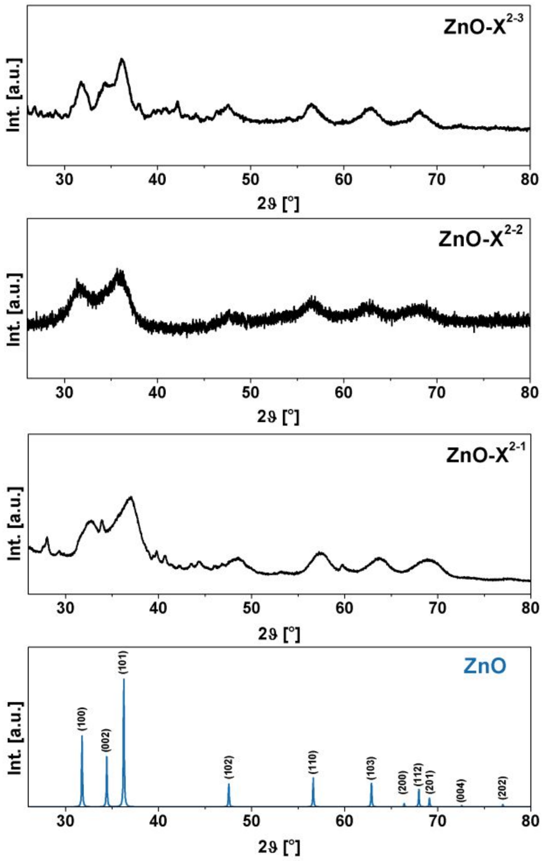

Figure S5. Powder X-ray diffraction pattern of ZnO-X$^{2-1}$ – ZnO-X$^{2-3}$ NCs in comparison with bulk ZnO pattern (blue line) confirming wurtzite crystalline core structure (note that no other ZnO-related crystalline phases are detected; the additional minor signals are most likely attributed to zinc carboxylate species).

Thermogravimetric analysis

ZnO-X^{2-1}

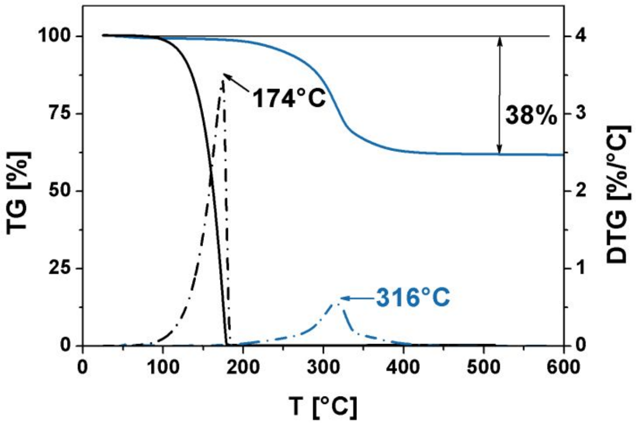

ZnO-X^{2-2}

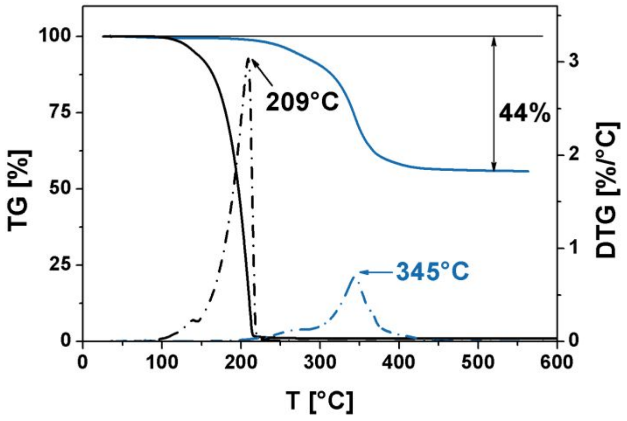

ZnO-X^{2-3}

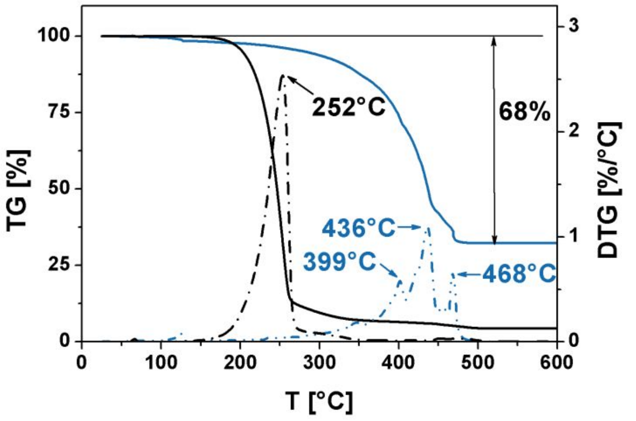

Figure S6. The TGA and derivative thermogravimetric analysis (DTG) traces showing the decomposition of ZnO-X^{2-1} - ZnO-X^{2-3} NCs (blue line) and the corresponding diorganophosphate pro-ligands (black line) in an air atmosphere.

Steady-state spectroscopy

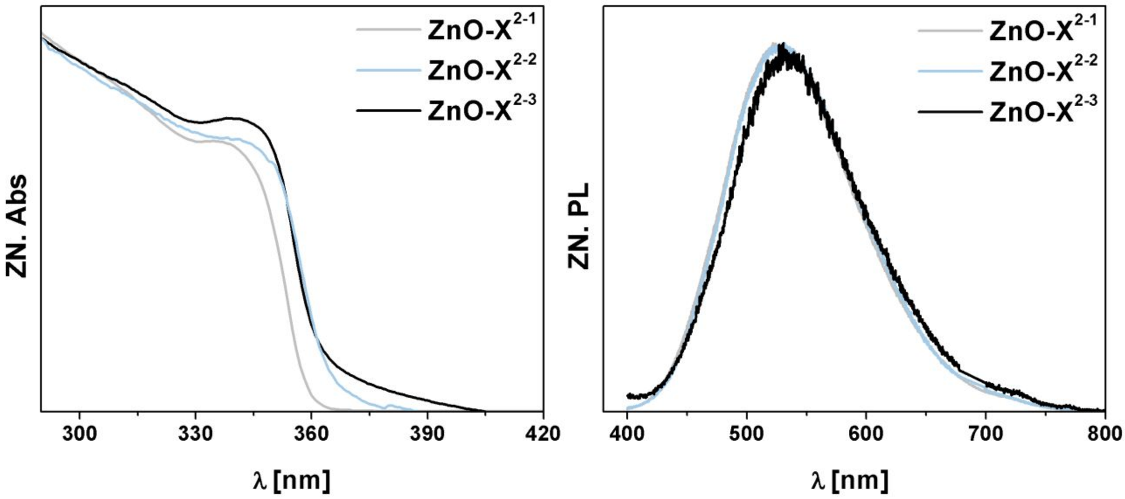

Figure S7. Normalized absorption (left) and emission (right) spectra of as-prepared reference-type carboxylate-coated ZnO NCs collected in THF.

OSSOM-derived NC's self-assembly at the air-water interface

Langmuir films of ZnO-X$^{1-0}$ NCs

Dimethyl phosphate ligand stabilizing ZnO-X$^{1-0}$ appeared too short to provide enough hydrophobicity. It was possible to record compression isotherms (Figure S8), but the films were not stable. The contact cross-sectional area (CCSA, see inset) did not scale with the added amount of colloidal suspension applied to the air/water interface. With the increasing volume of ZnO-X$^{1-0}$ suspension, the area should proportionally increase (as in the cases shown in Figure 3 in the main text). However, three times the applied volume resulted only in a small increase in the CCSA (inset in Figure S8). Compression caused the "injection" of ZnO nanocrystallites into the subphase. Consequently, this eliminated the ZnO-X$^{1-0}$ NCs from further investigations.

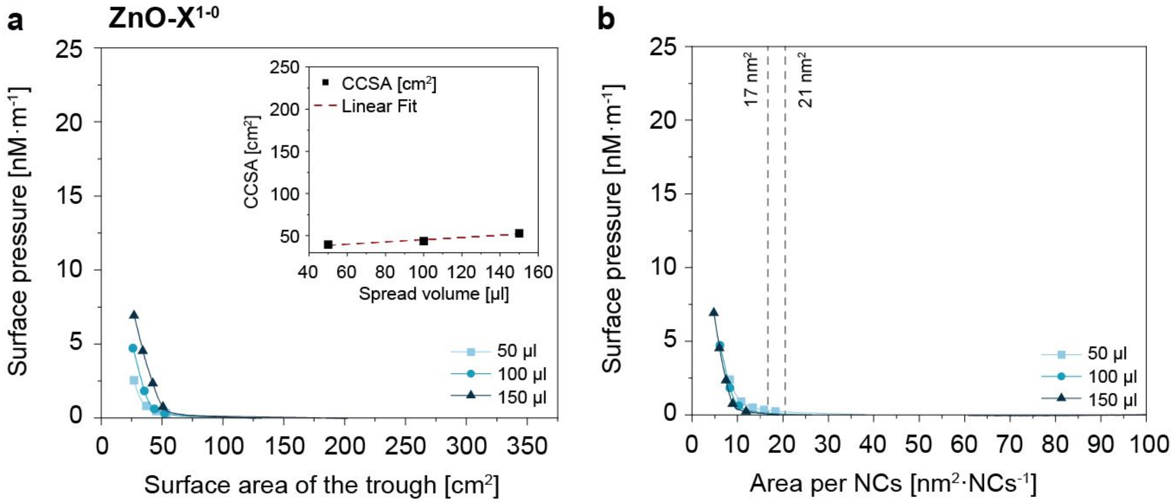

Figure S8. a) Surface pressure versus area isotherms and corresponding CCSA recorded for dimethyl phosphate-coated ZnO-X$^{1-0}$ NCs and b) compression isotherms expressed in a function of area per single ZnO NCs.

Reproducibility of Langmuir isotherms upon compression-decompression cycles

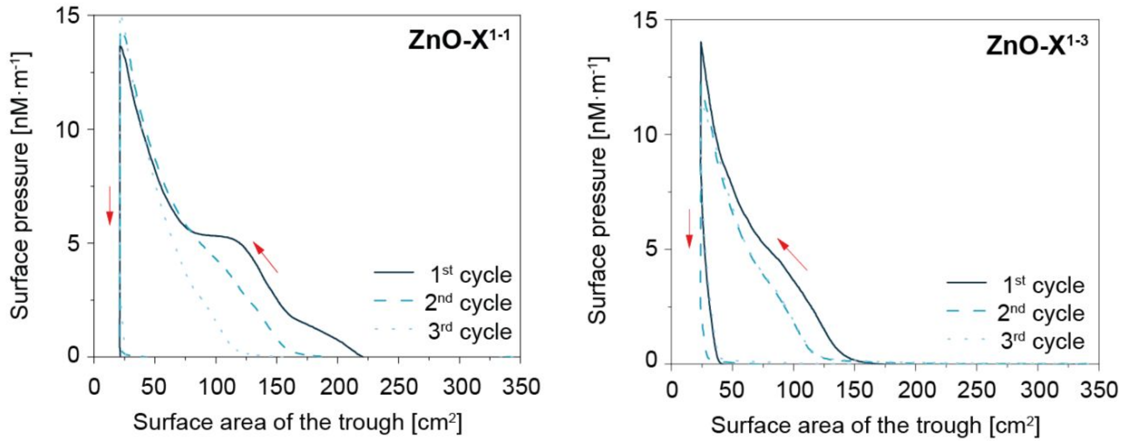

Figure S9. Reproducibility of Langmuir isotherms upon compression-decompression cycles.

Surface compressional modulus of Langmuir monolayers

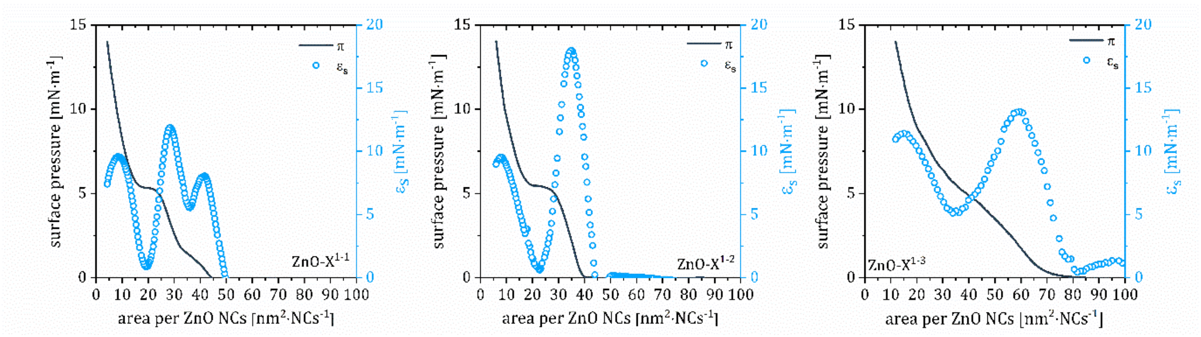

Figure S10. Surface compressional modulus as a function of area per single ZnO NCs.

Films transferred onto the solid substrates

Profilometry analysis

To assess whether the ZnO NCs form monolayers rather than multilayered aggregates, we compared key roughness parameters, including Total_Pa (mean roughness), Total_Pq (RMS roughness), Total_Psk (skewness), and Total_Pku (kurtosis), between the uncoated substrate and ZnO NC-coated films (ZnO-X^{1-1}, ZnO-X^{1-2}, and ZnO-X^{1-3}). The films were transferred according to the procedure described in the main text and used for SEM and XRR analysis. Profiles were measured using Dektak XT (Bruker Corporation, USA) with a 2.5 µm (radius) stylus.

The roughness values of ZnO-X^{1-1} and ZnO-X^{1-2} remain within a range comparable to the uncoated substrate, with Total_Pa values around 4 to 6 nm and Total_Pq around 5 to 7 nm. These modest increases in roughness support the formation of a relatively uniform NC monolayer rather than multilayered or highly aggregated structures. Importantly, such low roughness values measured across different locations of the film indicate that the films are homogeneous over the scanned areas and exclude the presence of highly uneven surfaces composed of large aggregates coexisting with thin regions, which would otherwise lead to greater variability in roughness. Such small values correspond to very smooth films, which is more likely for monolayers compared to films with plenty of 3D aggregates.

Due to the finite tip size and the nature of the measurement, each data point reflects an average over a small but non-negligible surface area. Therefore, the method is particularly suitable for detecting macroscopic or mesoscopic height variations. The lack of such variations supports the claim that the films were, in fact, flat layers, and due to SEM analysis – monolayers.

The Total_Psk (skewness) and Total_Pku (kurtosis) values for ZnO-X^{1-1} and ZnO-X^{1-2} remain close to those of a relatively smooth surface, further indicating that these films do not exhibit large-scale height variations or excessive roughness associated with multilayers. In contrast, ZnO-X^{1-3} exhibits significantly higher roughness (Total_Pa > 22 nm, Total_Pq > 47 nm) and extreme kurtosis values, indicating a rougher, more aggregated film.

These observations are well in line with SEM pictures in Figure 6 (main text).

The analysis confirms that ZnO-X^{1-1} and ZnO-X^{1-2} form well-defined monolayers, while ZnO-X^{1-3} is prone to aggregation. This supports our conclusions that ligand design critically affects monolayer formation. The profilometry measurements provide a robust validation of monolayer formation and align with the conclusions drawn from the compression isotherms and microscopy images.
